# Intra-Capsular Fractures of the Neck of the Femur

**Published:** 1895-12

**Authors:** G. A. Baxter

**Affiliations:** Chattanooga, Tenn.


### INTRA-CAPSULAR FRACTURES OF THE NECK OF THE FEMUR.

BY G. A. BAXTER, A.M., M.D.,

CHATTANOOGA, TENN.

The fracture is usually at the small part of the neck, though occasionally, in the very young, there may be a separation of the head. The line is more often transverse, but may be in any direction, transverse, oblique, irregular, and in one instance reported spiral. There may be interlocking of fragments, though this is very rare; when it does occur, it is the lower fragment into the upper one. Generally a large, smooth, convex surface presents on one fragment and a depression on the other. The head may be comminuted by the lower fragment.

The usual displacement is of the shaft upwards, backwards, and outwards, with or without laceration of the capsular ligament; however, this is varied in the proportion of one in every five cases to an upward and inward direction of the lower fragment. This displacement is a gradually increasing one, from one-half to one inch at first, increasing during the first two weeks to one and one-half to two inches.

The position of the limb is almost pathognomonic of this fracture; the foot widely everted, with shortening and a total inability (usually) to bear the slightest weight upon it, so that if the patient is in the upright position the body is supported on this side by the hands and not the leg. A further investigation will show an inability to lift the foot forward, as in the act of walking, or to raise the heel from the bed when lying down. In the impacted variety this may be modified to the extent of allowing the patient to stand upon the feet for a while, or to walk some distance before the leg gives way. There is pain, varying in character, but especially severe upon the slightest movement or pressure at the point of injury. There is a flattened condition of the hip on the injured side. Crepitus can generally be obtained by rotation inwards with slight extension to bring the fractured ends into apposition. This is not a constant symptom, and the search for it should always be carefully made, lest an irreparable injury be inflicted by unlocking a possible impaction. The trochanter rotates in a shorter radius, acting from the point of fracture rather than the head of the bone. It is above Nélaton's line in the triangular space described by Dr. Bryant, and nearer a line drawn in the axis of the body on the injured side (Morris's measurement).

Histologically the neck of the femur is constructed of cancellous tissue, surrounded by compact tissue, the latter varying in thickness at different parts of the neck as well as on its anterior and posterior aspects. On the anterior aspect the compact tissue is thinnest where it joins the head, and becomes thicker posteriorly, until it joins with the intertrochanteric line, which itself is compact tissue. On the posterior aspect of the neck the compact tissue is thickest where it joins the head, and becomes gradually thinner as it proceeds backwards and outwards, losing itself in the cancellous structure underneath the great trochanter, which is really an apophysis for the attachment of the external muscles, and adds little to the strength and resistance of the bone at this point. This makes the two weakest places in the bone just posterior to the head in the small part of the neck, where the anterior layer of compact tissue is thinnest, and at the base of the neck just above the trochanter, where the posterior layer grows thinnest before losing itself in the cancellous tissue underneath the great trochanter. These are the points at which fractures usually occur; hence we are forced to recognize these histological differences as predisposing causes to fractures at the upper end of the femur.

Beyond the age of fifty the bone undergoes a radical change in its structure, particularly at this point. The canals grow larger, the compact tissue thinner, the spongy tissues being filled with fat. The size is not altered, but the specific gravity becomes less and the resisting power of the bone to strain or force greatly diminished. This is not an absorption of animal tissue alone, but a senile atrophy; the changes which effect this condition being chiefly the enlargement of the canals and the thinning of the compact tissue, which latter becomes so thin at times as to be faintly translucent. Hence, we find that the great majority of these fractures occur beyond fifty.

Disease also, such as rheumatoid arthritis, contributes to or causes this fracture, though more often the head of the bone is absorbed in this disease in the hip-joint.

Another change, which occurs with age in both sexes, is an increasing acuteness in the angle between the neck and the shaft of the bone, the neck being found at times almost at a right angle to the shaft in the very old. The bone would undoubtedly stand a less degree of strain or violence with the neck at an acute angle with the shaft than at an oblique angle, but the fact of such change has been disputed by Rodet after investigations which led him to assert that the changes in the angle, as a result of age, were of so few degrees that as a causation it would have little effect. It is possible, however, that the difference in the angle between the sexes may explain, in part, the greater frequency of this fracture among women.

The exciting cause of fracture of the neck of the femur varies from the slightest degree of muscular strain transmitted through the limb from below, or in leverage, the limb being caught in adduction or abduction, to the greatest amount of direct force received directly upon the trochanter major. Stumbles, missteps, the jar received in stepping from the curbstone to the street, catching the foot in the carpet in turning or walking, loss of balance, twists of the body in emptying a pail of water, turning over in bed, bending to one side to keep from falling, falls on the knees, side of the thigh or on the buttock, muscular action in the movements of the thigh in adduction, abduction, or rotation, have all been held responsible for a degree of violence sufficient to produce the intra-capsular fracture. Malgaine asserted that exaggerated muscular action plays an important causative part at all times in the production of these fractures, in some cases the fall being secondary and in consequence of fracture produced by muscular action. The intra-capsular fracture more often occurs from the action of indirect force and lighter degrees of violence and in the aged and infirm; without the capsule occur more often in the young and healthy, and the exciting cause is more generally great and direct violence received directly on the trochanter, possibly transmitted from below.

The general question of repair of this fracture by bony union has been a subject of difference between surgeons; the difficulty of its satisfactory solution being a divergence of opinion in the interpretation of the processes which have taken place in the specimens of this fracture in existence. Owing to absorptive changes in the head and neck at the seat of fracture, it is said to be impossible to determine beyond cavil—1st. Whether the bone has been broken, or whether the changes are not simply the result of absorption. 2d. Whether, conceding a break, they were intra-capsular fractures purely, or, 3d. Whether the union is by fibrous or bony tissue. There can be little doubt that in early life, up to the age of seventeen years, there have been cases of repair by bony union in this fracture, but these cases are supposed by some to have been separation of the epiphyseal head, with impaction, not fractures, at the small part the neck, or those rare cases with little or no periosteum torn, and of consequently very little if any displacement of fragments. The great majority of surgeons of the present day claim that bony union takes place but very rarely, if at all, though close fibrous union does at times. The head of the bone, however, maintains life through a branch of the internal circumflex artery in the untorn portion of the periosteal covering, it being a fact that the periosteum is seldom torn completely around, the anterior-inferior portion escaping, and is capable of and does produce callus. In view of these facts, even barring those rarer cases of impaction which would undoubtedly have a better chance for union of this nature, even though the periosteum was completely around, for the cancellous structure itself has reproductive power of its own, it does seem that, with close and proper approximation and with absolute immobilization, it might be possible to get some kind of bony union here. The real difficulty unquestionably is, not the inability of the fragments to unite through defective vitality, but the heretofore impossibility of complete immobilization and the chance of approximation we were forced to accept as the best which could be done. The changes in the neck of the bone which predispose to fracture certainly do not of themselves decide this question in the negative, though, in any special case, either incomplete approximation, deficient immobilization, the completeness of the periosteal tear might do so. The attainment of close fibrous union of this fracture, giving a fair degree of usefulness to the limb, is an undoubted possibility. My own conclusions are, that could approximation and immobilization be maintained, there is nothing to prevent union by bone at this point, since, 1st. The upper fragment is alive and capable of granulation. 2d. Approximation heretofore, with most of the methods devised (extension and rotation inward or outward, as the case may require, for eversion or inversion of the foot), has been almost chance work, owing to our want of control of the upper fragment and its general displacement inwards by the pressure of the lower fragment, and our neglect of local control of the upper end of the lower fragment. These methods correct the upper and backward displacements, but they leave an outward displacement, due to the continued action of the hip muscles in this direction, still unconquered. Approximation being still incomplete, immobilization could do no good whatever. 3d. The rarer cases of reported union have been, must have been (with a chance approximation possible only), either cases where the periosteal covering was untorn or torn but slightly, cases of impaction, or cases of fracture partly within and partly without the capsular ligament.

Whatever the possibility in these cases, the result, in the past, has been an almost universally bad one—disability more or less, the patient suffering all degrees of functional interference, from permanent confinement in bed to the use of a crutch or cane, with close fibrous union and little deformity, but always with an amount of shortening from one-half to two inches.

The differentiation between intra- and extra-capsular fractures depends much upon the history of the case, the symptoms differing more in degree than in character, and it is asserted that an actual diagnosis between the two is a matter of little moment, since the treatment is practically the same in both varieties of fracture. Such reasoning should be condemned, as the hypothesis upon which it is based is untrue. The treatment for both these varieties of fracture need not necessarily be the same, or rather, the treatment which accomplishes a good result in the extra-capsular fracture has hitherto been an almost total failure in the intra-capsular, and there are points of differentiation of value for self-satisfaction and prognosis. Without being exhaustive, a few of these symptoms will be enumerated, that the contrast may be established for the purpose of precaution both in prognosis and treatment, as will be shown hereafter to be necessary.

The intra-capsular fracture is nearly always seen in the very old (fifty and upwards), while the extra-capsular fracture is more often seen before this period. The fracture within the joint is most common in women, while the fracture without the joint is equally common to both sexes. The degree of violence in the intra-capsular variety is usually very slight, which is seldom the case in the extra-capsular variety. The internal variety is produced from below (generally) by force transmitted through the limb, the external by force directly upon the trochanter. In the intra-capsular variety the shortening is small at first, gradually increasing with the progress of time; in the extra-capsular the shortening is immediate and complete. In the one case we have pain, redness, and swelling referred to the hip-joint, in the other to the trochanter. The only dislocation with which intra-capsular fracture would be likely to be confused is the pubic. This would give shortening and eversion, but the shortening would be greater and the eversion less in the dislocation. In intra-capsular fracture the displacement, if it exists, would be a drawing upwards, backwards, and outwards of the lower fragment, while in dislocation the head of the bone would be felt in the groin. In fracture we would have extra mobility with probable crepitus; in dislocation, diminished mobility and absence of crepitus. The pain existing in fracture would be ascribed to the joint; in dislocation, to the groin. In those exceptional cases where the foot is inverted in this fracture, the appearance of the hip alone (flattening) would indicate the impossibility of dislocation in the upward and backward direction. In any degeneration, as that of tuberculosis or rheumatoid arthritis, the history of previously existing disease of this character should lead to a proper conclusion.

*Treatment.*—The consideration of treatment involves the question of the possibility of union by bone at this point of fracture in the femur. An avowal of hopelessness on this point means helplessness, and it would become our duty, after a short interval for the subsidence of acute inflammatory symptoms, to abandon all restraint or confinement, instead of making a pretense of that which, under this supposition, can never be accomplished. But is such a supposition correct? Many surgeons, reasoning from the almost universal result, non-union, have jumped to the conclusion that there could be none, and have attributed the failures to a want of vitality in the upper fragment of the fractured bone. This may be, and doubtless is, the cause of non-union in some few cases where the violence has been very great, producing comminution of the upper fragment or tearing the periosteum completely around the whole circumference of the bone; but, that it is not universally the case is proven by the few cases of union of bone which have taken place in early life by the preservation of vitality in the upper fragment in all cases not comminuted, with even partially preserved periosteums, or uncomplicated with general conditions of disease tending to retard or defeat bone repair. It is further proved by the cases of cartilaginous union, as this is but the uncompleted process of union by bone. That the changes occurring as the result of age simply are not a hindrance to union is proven by the fact that the cases of union by fibrous tissue are not limited to the cases which occur in early life, but in old age as well.

It is a well-known fact that a fracture seldom occurs here so severe or extensive, that there is not some portion of the periosteum remaining untorn. This untorn portion is usually on the under and anterior aspect, and in it is rescued, for the nutrition of the upper fragment, one of the branches of the internal circumflex artery. This branch and the blood through the ligamentum teres are sufficient to give a healthy vitality to the detached head of the bone, and if it is ample to do this, it is equally plain that there should, under other proper conditions, be a production of callus. Some of the specimens of fibrous union in existence show a want of perfect approximation and a bridging over of an intervening space by fibrous union in spite of imperfect approximation, the fibers taking an oblique course rather than a straight direction with the axis of the neck portion of the lower fragment. This seems to prove that, with perfect approximation and retention, there would have been unquestionably bony union where fibrous union was accomplished in these cases, as the failure to preserve these two requisites (approximation and retention) is the main cause in the production of fibrous rather than bony union elsewhere.

The acknowledgment of the possibility of callous production between the fragments of this fracture leads to the confession of an attainable bony union, and we ask ourselves, what are the conditions required? To answer this question we must again inquire as to the displacement. In by far the greater number the displacement is upwards and backwards (shortening), with external rotation (eversion); in rare case upward and forward displacement and internal rotation; in either case the upper fragment is pushed to one side by the lateral pressure of the displaced lower fragment. In this condition, it is pertinent to ask, does extension and inward rotation (the correction of eversion and shortening), or extension and outward rotation in those rarer forms of displacement, correct the deformity entirely? Unquestionably they do overcome shortening and correct eversion by opposing gravity of the limb and counteracting the effect of the rotator muscles in twisting the limb, but they do not overcome the outer displacement, as made by such muscles as the gluteus medius on the upper end of the lower fragment, or bring it into apposition with the already partially displaced upper fragment, except it be by the merest chance; this chance being sufficient to account, together with impaction, for the very few cases of partial approximation as evidenced by ligamentous union. Evidently then, there is something more required in the treatment of this fracture than extension and rotation for its retention, which form the only principles maintained in nearly all the ordinary splints in common use for its correction. There needs must be, in addition to these principles, local internal pressure over the upper end of the lower fragment to bring it into relation with the upper fragment, besides continued pressure in line with the axis of the neck. The bulging trochanter seems the most convenient place to place such pressure, and it is possible to imagine this attained by any of the splints of the straight variety to which has been previously adjusted a pad for the trochanter, or with the suspension variety, with a pad or even sand-bag pressure. It is even possible that the sand-bags ordinarily with the latter variety, to keep the body straight, have by pressure, unconsciously to the surgeon, contributed towards a nearer approximation and consequently ligamentous union in a few cases. That surgeons of the past recognized something unattained is evidenced by the number of retention splints which have been devised, and that they were correct is proven by the almost total failure to obtain union by any of them. It remained for Dr. Nicholas Senn to correct this cause of non-union, and also to perfect the plaster splint so as to leave little to be desired in meeting the indications present. If we may hope for intra-capsular union at all, we must look for it through this apparatus or one fulfilling these indications equally as well.

I quote a description of this device from DaCosta: "To secure perfect immobility at the seat of fracture, it is not only necessary to include in the dressing the fractured limb and entire pelvis, but it is absolutely necessary to include the opposite limb as far the knee, and to extend the dressing as far as the cartilage of the eighth rib. The splint is incorporated in the plaster dressing, and it must be carefully applied, so that the compress, composed of a well cushioned pad with a stiff unyielding back, rests directly upon the trochanter major, and the pressure, which is made with a set-screw, is directed in the axis of the femoral neck. Lateral pressure is not applied until the plaster is completely set. Syncope should be guarded against by the administration of stimulants. As soon as the plaster has sufficiently hardened to retain the limb in proper position, the patient should be laid upon a smooth, even mattress, without pillows under the head, and in non-impacted fractures the foot is held in a straight position and extension is kept up until lateral pressure can be applied. No matter how snugly a plaster of Paris dressing is applied, as the result of shrinkage it becomes loose, and without some means of making lateral pressure, it would become necessary to change it from time to time in order to render it efficient. But by incorporating a splint in the plaster dressing, this is obviated and the lateral pressure is regulated day by day, by moving the screw, the proximal end of which rests on an oval depression in the center of the pad."

Concerning the final results in these cases but little more need be said. The results for good union in the past, as has been said, are practically negative. As to full restoration, it has been asserted that there never has been a proven case of full restoration with complete bony union, full length, natural position, and strength. While this is true and a large number of these cases remain permanent fixtures in bed, there is yet a large proportion who regain, under even the unfavorable conditions, a large amount of functional activity. Either the broken ends of the bones become smoothed by rubbing against each other, or the lower fragment makes for itself a new socket of fibrous tissue, and the outer fasciculus of the Y ligament and the tendon of the obturator support the weight of the body upon the shaft below. The permanent condition varies from that of bedridden helplessness to crutches or cane, but always with perceptible shortening and consequent lameness. I must enter my vigorous protest against the do-nothing policy so frequently followed in the very old, where, accepting death or non-union as inevitable, the patient is made as comfortable as possible under such a policy, in any position as to the limb, under the hopeless thought that it is all useless anyway. Such policy is worse than it seems without thought, for it contributes directly to the production of hypostatic pneumonia and fatty embolism, subjects the patient to tortures of continued confinement in bed and its concomitant results at this age—uncleanliness, bed-sore, and exhaustion, when all might be relieved by a proper application of the Senn device, at least until time had eradicated the immediate results of such an injury (active inflammatory action) and transferred him to that safer and less helpless state of non-union which has been the finale in these cases, thus giving to him at the same time whatever chance remained for him for the production of bony or fibrous union. With this splint the patient can be handled with impunity.

